# Supplementary material for: The activity of soil microbial taxa in the rhizosphere predicts the success of root colonization
Source: mSystems. 2025 Aug 6;10(9):e00458-25. doi: 10.1128/msystems.00458-25 (PMC12455943; doi:10.1128/msystems.00458-25)
Supplement: Supplemental Material — Supplemental methods, Fig. S1 to S8, and Tables S1 to S10. [file msystems.00458-25-s0001.pdf]

Supplementary Information

The activity of soil microbial taxa in the rhizosphere predicts the success of root colonization.

Submitted to: mSystem.

Jennifer E. Harris [a,b,c], Regina B. Bledsoe [a*], Sohini Guha [a], Haneen Omari [b**], Sharifa G. Crandall [d], Liana T. Burghardt [a,b], Estelle M. Couradeau [b, c] #

[a] Department of Plant Science, Penn State University, University Park, PA

[b] Ecology Program, Huck Institute for the Life Sciences, Penn State University, University Park, PA

[c] Department of Ecosystem Science and Management, Penn State University, University Park, PA

[d] Department of Plant Pathology and Environmental Microbiology, Penn State University, University Park, PA

# address correspondence to Estelle M. Couradeau, estelle@psu.edu

[*]Present address: Regina B. Bledsoe, NewLeaf Symbiotics, Inc., Saint Louis, MO

[**]Present address: Haneen Omari, RETI center, Brooklyn, NY

Liana T. Burghardt and Estelle M. Couradeau contributed equally to this work.

Supplemental Methods:

LC-MS soil metabolomics:

The natural level of methionine in soils was evaluated with LS-MS metabolomics. In three replicates, methionine from soils was extracted by adding 5 ml of LC-MS grade water to 1g of soil. Samples were incubated for 1 hour at 4C and vortexed for 5 seconds every 15 minutes. The samples were centrifuged at 3220xg for 15 minutes at 4C. We filtered the supernatant with a 0.45 µm filter and freeze-dried with Freezone 12L -50 Lyophilizer (Labconco) for 72 hours. Samples (5 µL) were separated by reverse phase HPLC using a Prominence 20 UFLCXR system (Shimadzu), with a Waters BEH C18 column (100mm × 2.1mm 1.7 um particle size) maintained at 55°C and a 20-minute aqueous acetonitrile gradient, at a flow rate of 250 µL/min. Solvent A was HPLC-grade water with 0.1% formic acid, and Solvent B was HPLC-grade acetonitrile with 0.1% formic acid. The initial conditions were 97% A and 3% B, increasing to 45% B at 10 min and 75% B at 12 min, where it was held at 75% B until 17.5 min before returning to the initial conditions. The eluate was delivered into a 5600 (QTOF) TripleTOF using a Duospray™ ion source (all Sciex). The capillary voltage was set at 5.5 kV in positive ion mode with a declustering potential of 80V. The mass spectrometer was operated with a 100 ms TOF scan from 50 to 500 m/z and 16 MS/MS product ion scans (100 ms) per duty cycle using a collision energy of 50V with a 20V spread. Then, the Penn State Huck Metabolomics core quantified methionine peaks in Sciex Analyst software.

Probing Microbial Activity with BONCAT:

We used BONCAT to label metabolically active cells in bulk soil, rhizosphere, root endosphere, and nodules. After incubation with HPG and cell extraction, a copper-catalyzed click reaction was performed to attach FAM picolyl azide dye (Ex/Em 490/510 nm) to the newly made proteins during the greenhouse incubation. We followed the protocol described in Couradeau et al. 2019 (full detailed protocol in supplemental methods. (Briefly, 700 µl of each cell extract was captured on a 0.22 µm Millipore isopore filter, and the liquid was removed by vacuum filtration. Then, the sample-containing filter was transferred to a glass microscope slide, where we added 80 µl of the reaction mix. The reaction mix consisted of 5 µl copper sulfate ($CuSO_4$ 100 µM final concentration), 10 µl tris-hydroxypropyltriazolylmethylamine (THPTA, 500 µM final concentration), and of 3.3 µl (FAM picolyl azide

dye, 5 μM final concentration), buffered in of 50 μl of 5 mM sodium ascorbate freshly prepared in 1M PBS and 50 μl of 5 mM aminoguanidine HCl freshly prepared in 1M PBS and 880 μl of 1M PBS. All reagents were purchased from Click Chemistry Tools. The sample-containing filter was sealed with a cover slip and incubated in a dark place at room temperature for 30 minutes. After the incubation, the excess dye was washed off by soaking the sample PBS for five minutes three times. Cells were removed from the filter by vortexing in PBS-tween for 5 min. Finally, we added SYTO59 DNA counterstain (Invitrogen, Ex/Em 622/645 nm) at a final concentration of 0.5 μM.

Flow cytometry gating and sorting:

BONCAT fluorescence was first evaluated on a BD LSRFortessa Cell Analyzer (Becton, Dickinson, and Company). The threshold size was set to above 0.2 μm determined by Flow Cytometry Sub-Micron Particle Size Reference Kit Beads (Invitrogen). BD LSRFortessa Cell Analyzer was set up to capture the FAM picolyl azide dye (Ex/Em 490/510 nm) in the green channel of a 488 nm blue laser and the SYTO59 DNA counterstain (Ex/Em 622/645 nm) in the red channel of a 630 nm red laser. Viable cells were identified by drawing a SYTO59-positive gate against an unstained control. Active cells were gated from the SYTO59-positive events using a nested gating strategy. Active cells were determined by BONCAT FAM picolyl azide-positive events against a water-incubated clicked control to account for the background fluorescence from the click reaction. The false positivity recovery rate was capped at 0.2% in the gating process. We used the same gating strategy for Fluorescence-activated cell sorting (FACS) on a MoFlo Astrios Cell Sorter (Beckman Cloutier) to sort cells from plant compartments (rhizosphere, root, nodule). We could not recover enough active cells from the bulk soil samples due to their low cell event numbers and low activity to include them in the downstream process. We sorted from each sample (mean 82,000) for the active cell fraction, and only samples with at least 50k Active cells were included in our analysis (see Supplementary Table 2 for cells sorted counts from each sample). For the viable cell fraction, we sorted 100k Viable cells from each sample, using unclicked cell extract and directly labeled with a final concentration of 0.5 μM of SYBR Green (Invitrogen, Ex/Em 498/522 nm) as a general DNA probe.

DNA extractions and 16S rRNA amplicon sequencing:

We sequenced five plants' active and viable fractions from three compartments (rhizosphere, root endosphere, and nodule). We also sequenced Total DNA from Bulk Soil and rhizosphere (four replicates). For active and viable fractions, our library prep protocol was adapted from Reichart et al. 2020 [32]. Cells were centrifuged at 14000g for 10 min at 4°C. The supernatant was pipetted out, and 9.2 µl of nuclease-free water was added. Cells were then lysed with prepGEM® Bacteria Kit cell lysing kit following manufacturer instructions (New England Biolabs). We extracted total DNA from all rhizosphere and bulk soil samples with the DNeasy PowerSoil Pro Kit (Qiagen). We used 515f-Y (GTGYCAGCMGCCGCGGTAA, Parada 2016) and 806R-B GGACTACNVGGGTWTCTAAT, April 2015) primers to amplify the 16S rRNA gene from lysed cells pellets and total DNA. Our final PCR reaction mixture included a final concentration of 1x Invitrogen Platinum Super Master Mix and .2 µM of each primer. The thermocycler program included an initial denaturation of 98C for 2 minutes, 30X cycles of 98°C for 10 seconds, 56.2°C for 20 seconds, 72°C for 15 seconds, and a final elongation of 72°C for 5 minutes. The Huck Life Science Genomics Core conducted library preparation and indexing. Of the 30 samples (2 fractions X 3 compartments X 5 reps) we sorted cells from, 27 samples had successful library preparation, resulting in at least four complete reps for each treatment. The successful libraries were sequenced on an Illumina MiSeq platform using 250 bp x 250 bp paired-end sequencing, aiming at 100,000 reads per sample. The sequencing run produced 7,187,623 reads, averaging 107,255 paired reads per sample.

Microscopy:

We used confocal microscopy to visualize BONCAT-labeled active microbial cells in the nodule. Pre-inoculated *Trifolium incarnatum* was grown in potting soil in the greenhouse. After eight weeks, three plants were dosed with HPG dissolved in water at a final concentration of 0.038 µmol HPG per g soil, and one plant with HPG-free water as a negative control. After 24 hours, nodules were picked, washed, and stored in PIPES buffer for ~16 hours. Next, nodules were embedded nodules in Epredia Cryomatrix resin and frozen. 50 µm thick sections were cut from distal to proximal end onto poly-lysine-coated slides with a Leica CM1950 Cryostat. After sectioning, the cryomatrix was dissolved by soaking sections in DI water for 5 minutes. The BONCAT reaction mix was the

same as our flow cytometry samples. However, we substituted Picolyl-Azide-5/6-FAM for Cy3 Picolyl Azide (Click Chemistry Tools, Ex/Em 553/568) because plant cell walls showed the least autofluorescence in this Ex/Em range. 100 µl of BONCAT reaction mix was added to each section and incubated for 1 hour at room temperature in the dark. After incubation, excess dye was removed by washing with PBS three times. BONCAT labeled sections were imaged on the Leica SP8 DIVE multiphoton microscope. BONCAT Cy3 dye was excited on a diode 553-laser line and captured fluorescence with sensitive hybrid detectors (HyDs). For visualizing the general nodule structure, sections were incubated for 5 minutes with .1% toluidine blue. Toluidine blue images were captured on a Zeiss Axio Observer fluorescence microscope with an Axio 208 color camera. Images were processed using ImageJ (Fiji) [31]. Using BONCAT and toluidine blue, we could identify zones of nodules with metabolically active microbial cells.

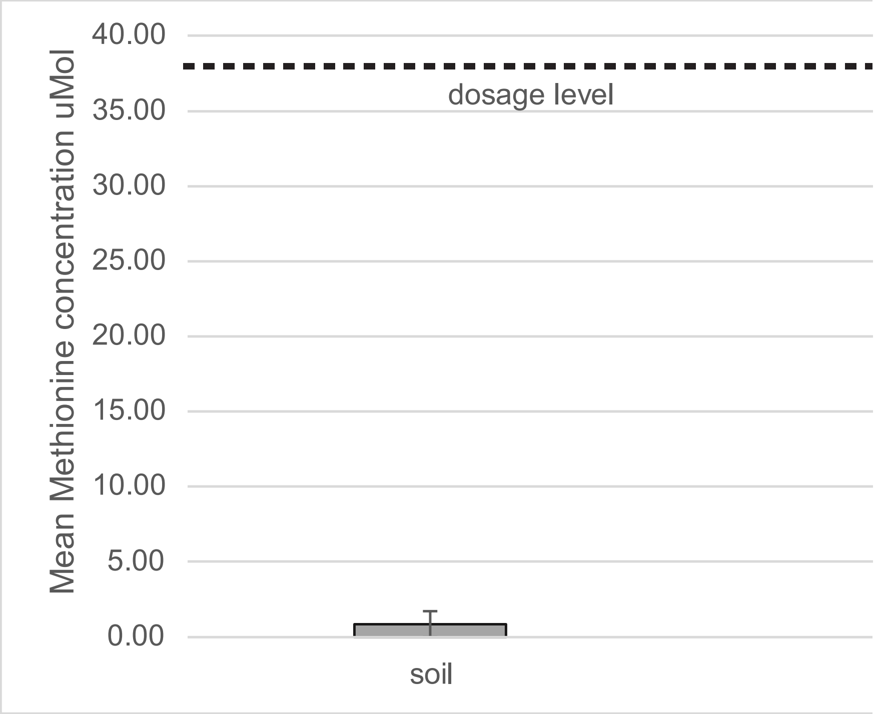

Supplementary Figure 1: Mean natural methionine concentrations from soil used in the greenhouse experiment in µM, measured with LC-MS. Bar indicates the mean and error bars are standard deviation (n=2; one sample was below the detection limit). The dashed line shows the HPG dosage level. Concentrations were calculated from a standard curve of known concentrations of methionine. The methionine peak area was adjusted by the area of an internal standard (Chlorpropamide).

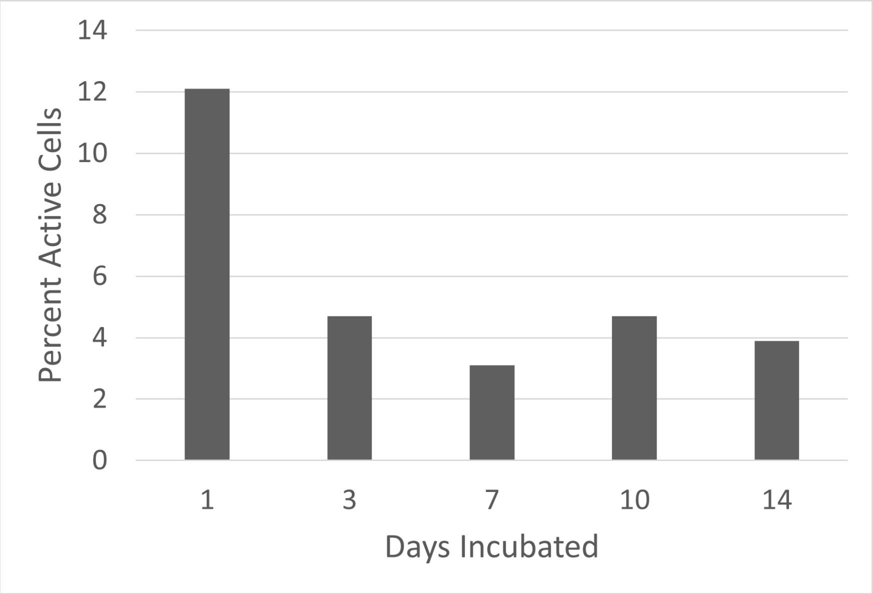

Supplementary Figure 2: BONCAT labeling of active cells for increasing HPG incubation times. Soils were incubated with 50 µl of HPG / gram of soil at room temperature. Percent Active Cells is the proportion of BONCAT active cells to viable cells, measured by flow cytometry.

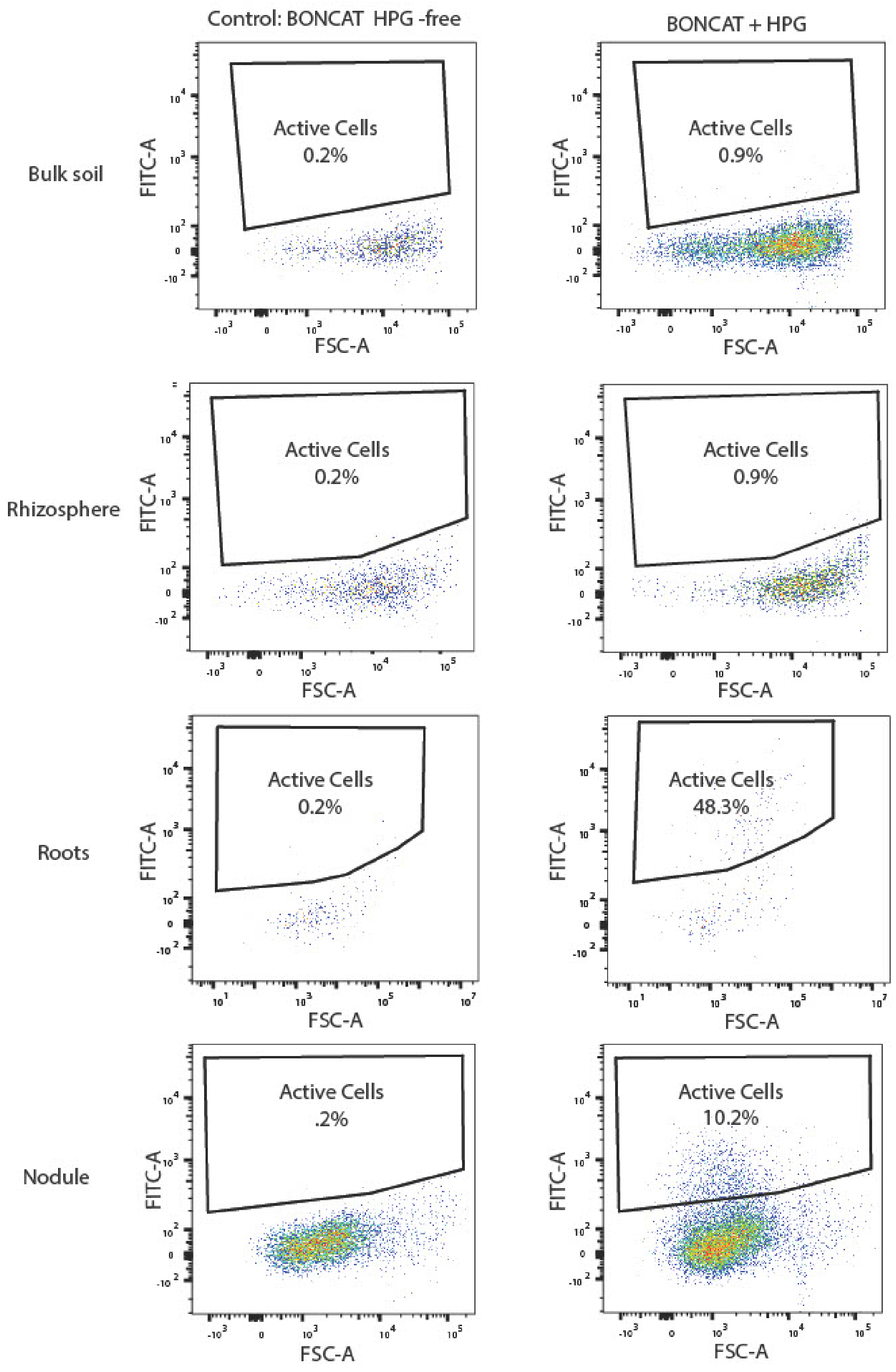

Supplementary Figure 3: Flow Cytometry of BONCAT labeled cells in bulk soil, rhizosphere, roots, and nodules. All events shown are SYTO positive. Flow cytometer events are plotted using their forward scatter (FSC, x-axis) and BONCAT fluorescence (FITC, y-axis) as a log-log scale. Increased cell event density is indicated by color. The BONCAT+ active cells gate is displayed as a box in each plot and labeled with the percent BONCAT+. Water-incubated controls treated with the BONCAT reaction mix are in the left column, then HPG-incubated samples treated with the BONCAT reaction mix are in the right.

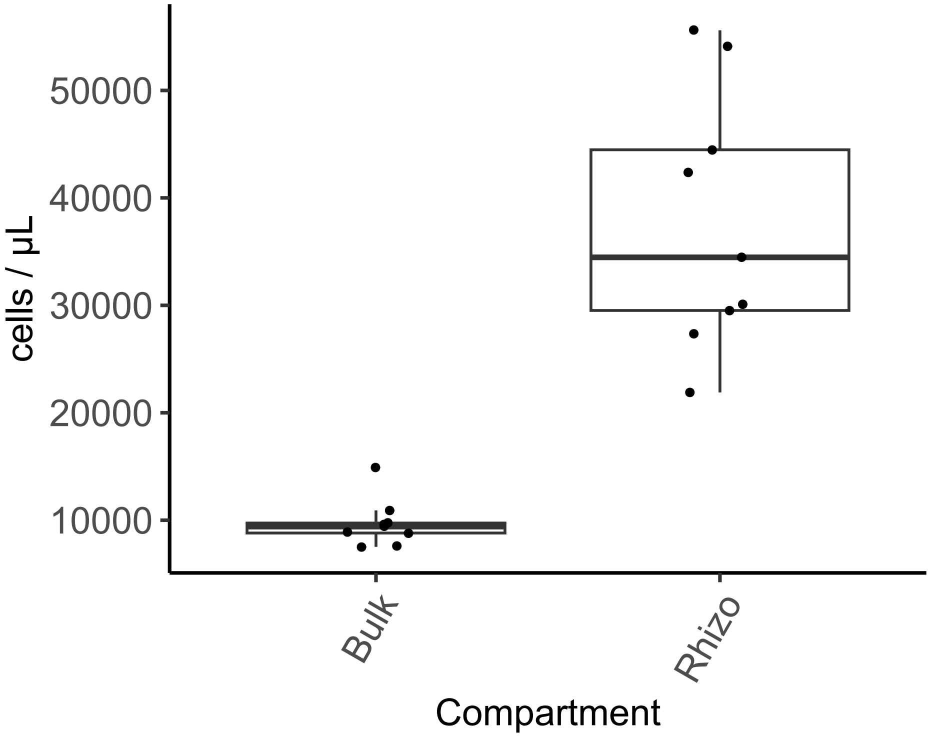

Supplementary Figure 4. The cell counts per microliter of sample for bulk soil and rhizosphere. Cell counts were collected on a Macsquant VYB flow cytometer (Miltenyi Biotec). Cells were identified as SYBR green positive against an unstained control. The rhizosphere has significantly more cells than bulk soil in a linear model (cell/µl ~ compartment, df = 16, $p < 0.001$, $R^2 = 0.7334$).

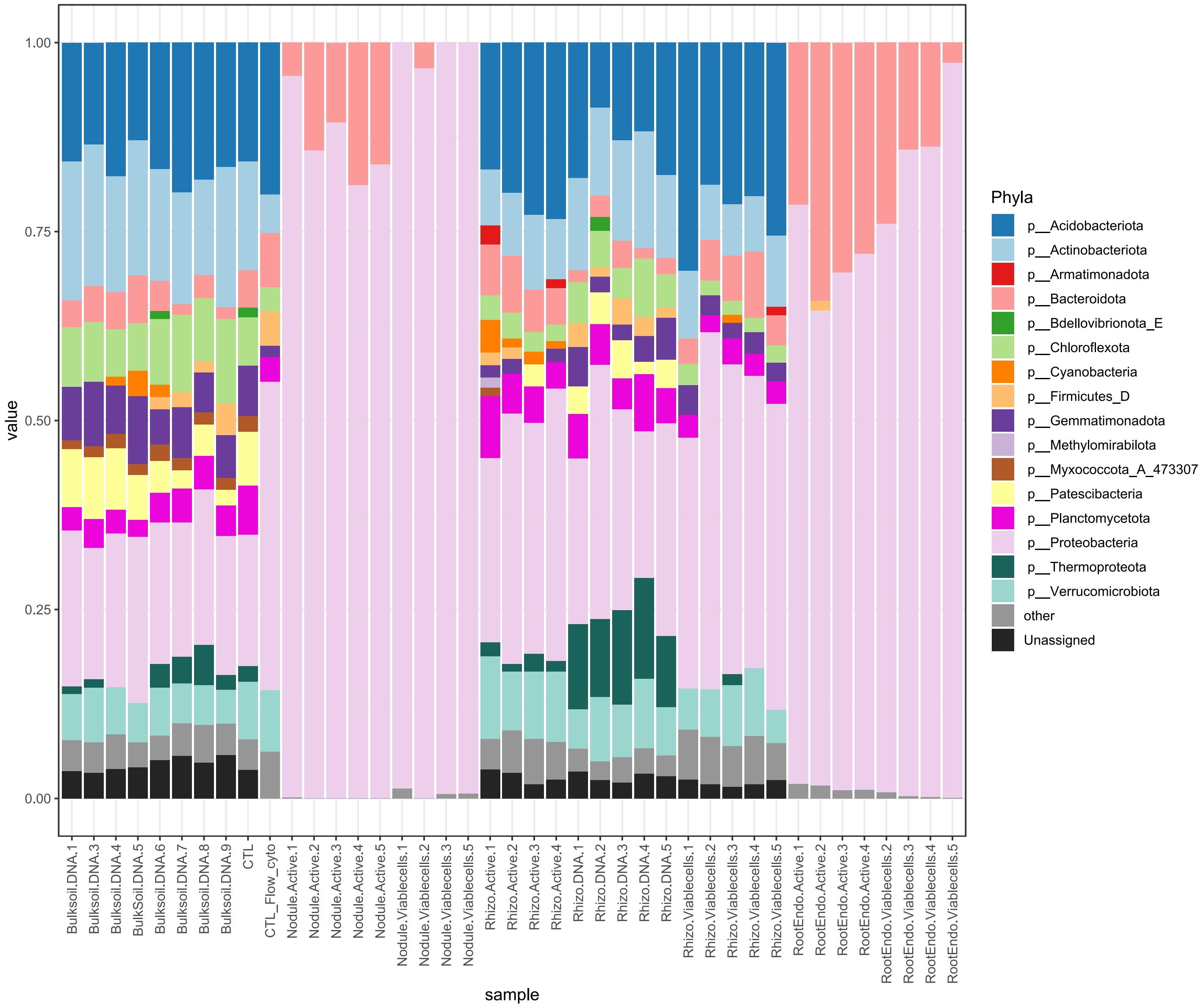

Supplementary Figure 5: Phyla level microbial community composition across all samples. Each stacked bar is a sample. Sample names are on the X-axis. Taxa with less than 1% abundance were placed in “other.” ASVs are unrarefied.

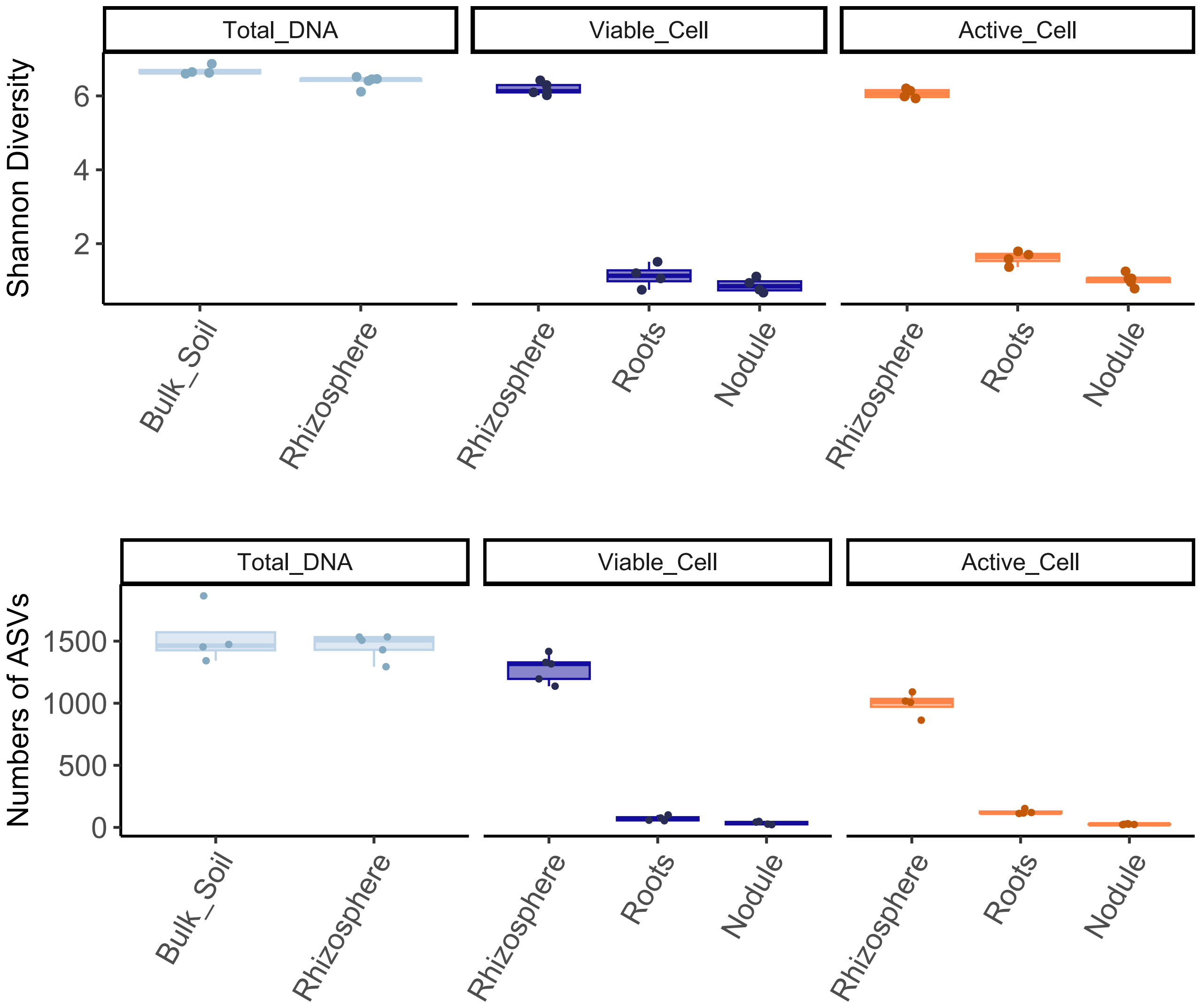

Supplementary Figure 6: Shannon Diversity calculated from unrarefied reads (top). ASVs richness from unrarefied reads (bottom). Replicates are from different plants (n=4).

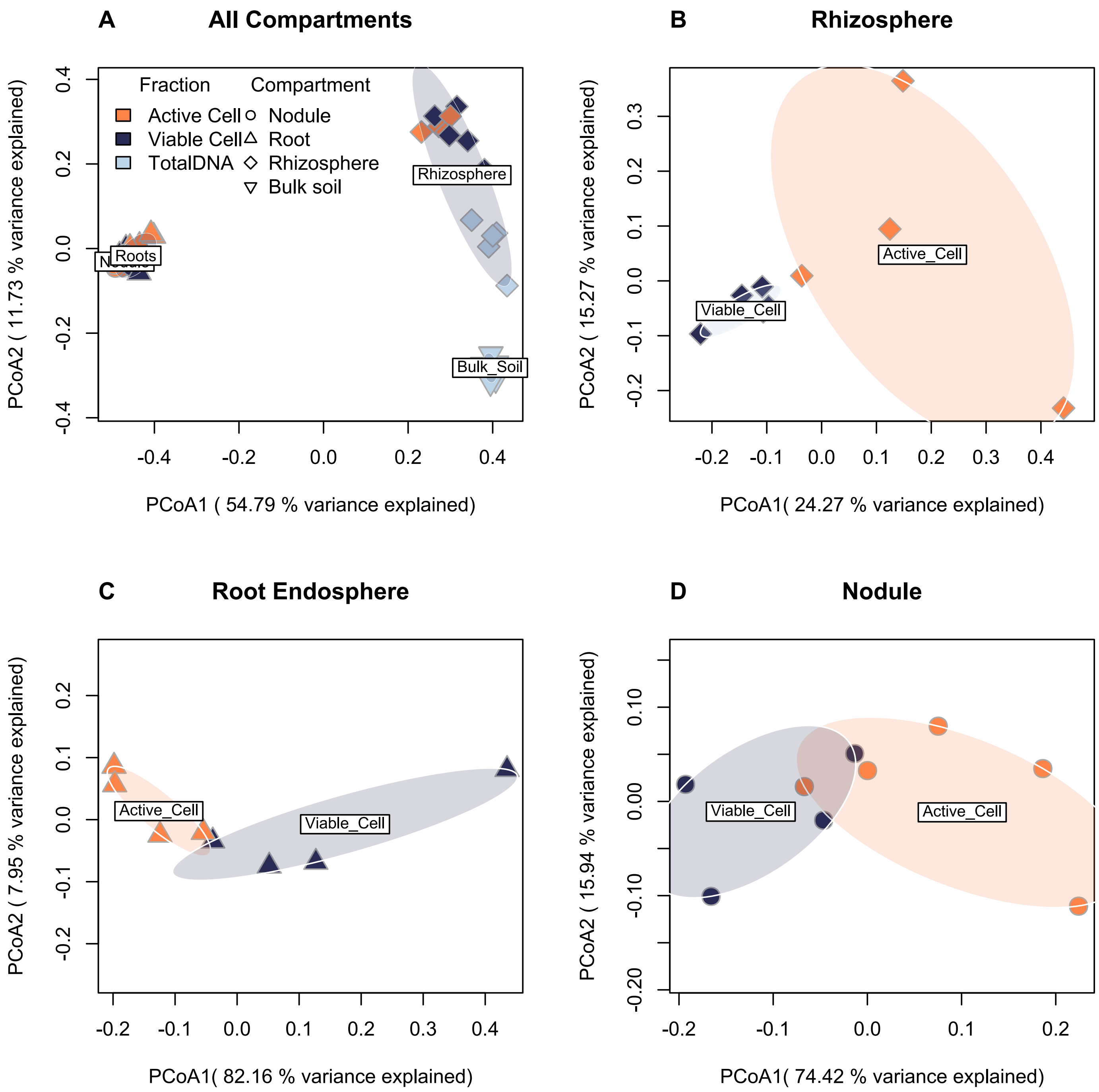

Supplementary Figure 7: PCoA of Bray Curtis dissimilarities of microbial communities on unrarefied reads from ASVs of 16S rRNA. Ovals represent 95% confidence intervals. A) Total dataset showing microbial communities across compartments and fractions. B) Rhizosphere active and viable communities do not overlap in their 95% confidence interval in C) Root Endosphere and D) Nodule. Colors indicate the fraction sampled; shapes indicate the compartment sampled.

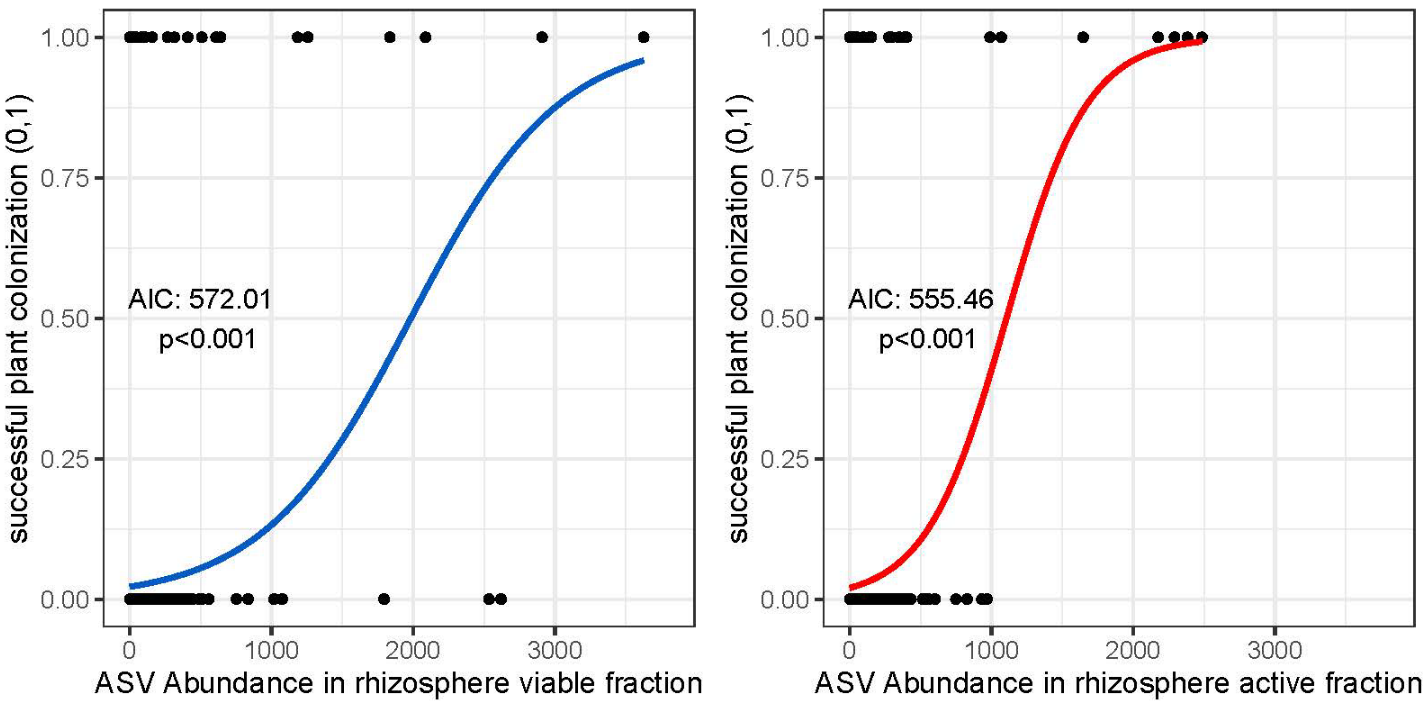

Supplemental Figure 8: Successful plant colonization is more associated with abundance in the active fraction than the viable fraction. Plots visualize the binomial model of the association between ASV abundance in the rhizosphere viable fraction (shown in blue) or in the rhizosphere active fraction (shown in red) with successful plant colonization. An ASV has successful plant colonization if it has at least 50 reads across all root or nodule endosphere compartments in either fraction. Model AICs and P-values are shown on the charts. Both models used the rarefied, filtered dataset.

Supplementary Table 1: Likelihood ratio test results of the ratio of active cells to remaining viable cells in a quasibinomial model ((number of active cells, remaining viable cells) ~ fraction + rep). Data was collected on the BD Fortessa flow cytometer.

Comparison	*df*	*Deviance*	*Resid.df*	*Resid. Deviance*	*P value*	
Bulk soil vs. Rhizosphere	1	16.3	12	95.7	0.15	
Bulk soil vs. Roots	1	3635	12	2016	3.30E-05	*
Bulk soil vs. Nodule	1	3953	12	1895	1.67E-05	*
Rhizosphere vs. Roots	1	2997	12	1986	1.57E-04	*
Rhizosphere vs. Nodule	1	3125	12	1865	1.24E-04	*
Roots vs. Nodule	1	12.2	12	3785	0.86	

*Significant in the model (p<.05)

Supplementary Table 2: 16S rRNA samples description, numbers of cells sorted, and reads passing quality control, number of unrarefied ASVs, and number of ASVs when rarefied to 40592 reads. CTL_S66 is a PCR control of water; BEADS is a flow cytometer sorting fluid. Sample *C7E-POS_S34** was omitted from the analysis because too few cells were collected.

Sample ID	*Compartment*	*Fraction*	*Replicate Pot*	*No. Cells sorted*	*Raw Reads*	*Reads passing QC*	*No. ASVS*	*No. ASVS rarefied*
C10N-POS_S65	Nodule	Active	1	60732	159315	138515	24	23
C1N-POS_S61	Nodule	Active	2	70966	153712	92476	25	24
C2N-POS_S62	Nodule	Active	3	66599	173557	135988	26	23
C5N-POS_S63	Nodule	Active	4	100000	188756	130178	22	22
C7N-POS_S64	Nodule	Active	5	100000	138863	100742	30	29
C10R-POS_S30	Rhizosphere	Active	1	97,888	101997	83847	864	855
C1R-POS_S27	Rhizosphere	Active	2	100000	105336	80724	1091	1086
C2R-POS_S28	Rhizosphere	Active	3	100000	91265	67920	1017	1014
C5R-POS_S29	Rhizosphere	Active	4	100000	88102	61804	1007	1005
C10E-POS_S60	Root	Active	1	50506	147464	85169	116	114
C1E-POS_S31	Root	Active	2	58919	145971	82832	152	147
C2E-POS_S32	Root	Active	3	73549	154251	76644	112	109
C5E-POS_S33	Root	Active	4	100000	175034	100352	120	114
*C7E-POS_S34**	Root	Active	5	21364	227295	142404	150	141
C10N-SYBR_S26	Nodule	Viable Cell	1	100000	188796	170390	49	47
C1N-SYBR_S13	Nodule	Viable Cell	2	100000	234920	180861	42	37
C2N-SYBR_S15	Nodule	Viable Cell	3	100000	125690	114107	26	24
C7N-SYBR_S25	Nodule	Viable Cell	5	100000	153058	130912	22	21
C10R-SYBR_S20	Rhizosphere	Viable Cell	1	100000	78509	48992	1137	1137
C1R-SYBR_S16	Rhizosphere	Viable Cell	2	100000	121003	85773	1329	1320
C2R-SYBR_S17	Rhizosphere	Viable Cell	3	100000	126308	87086	1317	1309
C5R-SYBR_S18	Rhizosphere	Viable Cell	4	100000	103848	68636	1417	1410
C7R-SYBR_S19	Rhizosphere	Viable Cell	5	100000	95994	62332	1196	1194
C1E-SYBR_S21	Root	Viable Cell	2	100000	157198	105567	100	97
C2E-SYBR_S22	Root	Viable Cell	3	100000	154515	103885	76	75
C5E-SYBR_S23	Root	Viable Cell	4	100000	161291	117193	58	54
C7E-SYBR_S24	Root	Viable Cell	5	100000	217885	193245	54	46
C10B-DNA_S4	Bulk Soil	Total DNA	1	NA	82320	40592	1342	1342
C2B-DNA_S1	Bulk Soil	Total DNA	3	NA	103445	46386	1454	1452
C5B-DNA_S2	Bulk Soil	Total DNA	4	NA	109593	59049	1865	1859
C7B-DNA_S3	Bulk Soil	Total DNA	5	NA	90895	49548	1474	1472
S10-DNA_S9	Bulk Soil	Total DNA	6	NA	107278	59880	1955	1945
S2-DNA_S5	Bulk Soil	Total DNA	7	NA	80802	48575	1551	1549
S3-DNA_S6	Bulk Soil	Total DNA	8	NA	92770	49128	1645	1640
S8-DNA_S7	Bulk Soil	Total DNA	9	NA	83071	50225	1539	1537
C10R-DNA_S12	Rhizosphere	Total DNA	1	NA	112015	58573	1430	1413
C1R-DNA_S8	Rhizosphere	Total DNA	2	NA	101059	50926	1294	1292
C2R-DNA_S10	Rhizosphere	Total DNA	3	NA	114438	62767	1533	1514
C5R-DNA_S11	Rhizosphere	Total DNA	4	NA	103825	67223	1534	1521
C7R-DNA_S14	Rhizosphere	Total DNA	5	NA	123393	67185	1507	1484
CTL_S66	Control	Control	NA	NA	104767	63774	1642	1640
BEADS	Control	Control	NA	NA	92886	72911	815	812

Supplementary Table 3: ANOVA comparisons for Shannon diversity of microbial communities. We subset data into fractions to compare Shannon diversity across compartments (Shannon diversity ~ compartment). We subset data into compartments to compare the diversity between active and viable fractions. Shannon diversity is calculated on rarefied reads in R package phyloseq.

Comparison[1]	*df*	*Effect Size*	*Standard Error*	*t value*	*p-value*	
Total DNA: Bulk Soil vs. Rhizosphere	7	-0.30	0.10	-3.1	0.018	*
Viable Cells: Rhizosphere vs. Roots	7	-5.06	0.16	-31.1	9.1E-09	*
Viable Cells: Roots vs. Nodule	6	-0.26	0.19	-1.4	0.212	
Active Cells: Rhizosphere vs. Roots	6	-4.44	0.11	-40.2	1.6E-8	*
Active Cells: Roots vs. Nodule	7	-0.60	0.12	-5.2	1.3E-03	*
Rhizosphere: Total DNA vs. Viable Cells	8	-0.20	0.10	-1.9	0.082	
Rhizosphere: Total DNA vs. Active Cells	7	-0.33	0.10	-3.3	0.012	*
Rhizosphere: Active vs. Viable cells	7	0.13	0.10	1.3	0.248	
Roots: Active vs. Viable Cells	6	-0.49	0.18	-2.7	0.037	*
Nodule: Active vs. Viable Cells	7	-0.15	0.12	-1.2	0.263	

*Significant in the model (p<.05)
[1]Reference level/group listed first

Supplementary Table 4: ANOVA comparisons for the number of ASVs microbial communities. We subset into fractions to compare the No. ASVs detected across compartments (No. ASVs ~ compartment). We subset data into each compartment to compare between active and viable fractions. No. ASVs are from rarefied reads.

Comparison[1]	*df*	*Effect Size*	*Standard Error*	*t value*	*p-value*	
Total DNA: Bulk Soil vs. Rhizosphere	7	-86	110	-0.8	0.457	
Viable Cells: Rhizosphere vs. Roots	7	-1206	55	-21.6	1.2E-07	*
Viable Cells: Roots vs. Nodule	6	-36	13	-2.8	0.033	*
Active Cells: Rhizosphere vs. Roots	7	-965	43	-22.7	8.3E-08	*
Active Cells: Roots vs. Nodule	7	-97	8	-12.4	5.1E-06	*
Rhizosphere: Total DNA vs. Viable Cells	8	-170.8	65	-2.6	0.030	*
Rhizosphere: Total DNA vs. Active Cells	7	-455	65	-7.0	2.0E-04	*
Rhizosphere: Active vs. Viable cells	7	284	70	4.1	4.7E-03	*
Roots: Active vs. Viable Cells	6	-53	14	-3.7	0.010	*
Nodule: Active vs. Viable Cells	7	8	6	1.5	0.185	

*Significant in the model (p<.05)
[1]Reference level/group listed first

Supplementary Table 5: ANOVA comparisons for evenness of microbial communities. We subset into fractions to compare evenness across compartments. We subset data into each compartment to compare active and viable fractions. Evenness is calculated using rarefied reads using the formula Evenness = Shannon diversity/ (Natural log(Observed ASVs))

Comparison[1]	*df*	*Effect Size*	*Standard Error*	*t value*	*p-value*	
Total DNA: Bulk Soil vs. Rhizosphere	7	-0.01	0.01	-4.5	2.7E-03	*
Viable Cells: Rhizosphere vs. Roots	7	-0.60	0.03	-21.9	1.1E-07	*
Viable Cells: Roots vs. Nodule	6	-0.01	0.04	-0.3	0.788	
Active Cells: Rhizosphere vs. Roots	7	-0.54	0.02	-30.8	7.8E-08	*
Active Cells: Roots vs. Nodule	7	-0.02	0.03	-0.6	0.569	
Rhizosphere: Total DNA vs. Viable Cells	8	-0.01	0.01	-1.0	0.353	
Rhizosphere: Total DNA vs. Active Cells	7	7E-04	0.01	0.1	0.939	
Rhizosphere: Active vs. Viable cells	7	-0.01	0.01	-1.0	0.348	
Roots: Active vs. Viable Cells	6	-0.07	0.03	-2.2	0.073	
Nodule: Active vs. Viable Cells	7	-0.06	0.04	-1.7	.131	

*Significant in the model (p<.05)
[1]Reference level/group listed first

Supplementary Table 6: Permutation test results of groups beta dispersion. Beta dispersion was calculated on groups' Bray Curtis dissimilarity matrixes. We did a permutation test of the groups' beta dispersion to determine if the variance was homogeneous between groups, which is required for a PERMANOVA.

Comparison	*Df total*	*Sum sq. total*	*Mean sq. total*	*F value*	*No. of Permutations*	*p Value*	
Full community Between Compartments	38	0.737	0.214	71.02	999	0.001	*
Bulk soil + Rhizosphere: Between Compartments	21	0.063	0.026	12.55	999	0.001	*
Rhizosphere: All fractions	13	0.040	0.017	29.28	999	0.001	*
Rhizosphere: Active vs Viable Cells	8	0.018	0.014	19.80	999	0.001	*
Total DNA: Bulk Soil vs Rhizosphere	8	0.004	0.001	2.30	999	0.166	
Roots + Nodule: Between Compartments	16	0.075	0.014	2.27	999	0.131	
Roots + Nodule: Active vs. Viable Cells	16	0.108	0.010	0.43	999	0.511	
Roots: Active vs. Viable Cells	7	0.031	0.005	0.05	999	0.884	
Nodule: Active vs. Viable Cells	8	0.023	0.004	0.30	999	0.592	

*Significant value (p<.05) indicates nonhomogeneous variance between groups.

Supplementary Table 7: PERMANOVA test results calculated on Bray Curtis distances. The full data set was a subset to evaluate differences between compartments or fractions.

Comparison	*Df total*	*Sum Sq total*	R^2	*F value*	*p-value*	
Bulk soil + Rhizosphere: Comparing Total DNA fractions	21	1.20	0.26	7.14	0.001	*
Roots + Nodule: Between Compartments	16	0.60	0.36	8.26	0.008	*
Roots + Nodule: Between Fractions	16	0.60	0.29	6.16	0.028	*
Roots: Between Fractions	7	0.26	0.60	8.92	0.032	*
Nodule: Between Fractions	8	0.13	0.51	7.30	0.028	*

*Significant differences between groups (p<.05)

Supplementary Table 8: Differentially abundant ASVs in the rhizosphere determined by ANCOM-BC. Log fold change reference is the viable fraction. A positive log fold change indicated that ASVs were enriched in the active fraction. We reported the lowest taxonomic classification identified with the greengenes2 database. Column name abbreviations are: LFC-log fold change viable to active community; Std. Error- standard error of the log fold change. Viable – mean number of rarefied reads per sample in rhizosphere viable fraction; Active – mean number of rarefied reads per sample in rhizosphere viable fraction. Adj. P value – adjusted p value from ANCOM-BC which controls for the FDR of multiple testing.

ASV	*Phyla*	*Lowest taxonomic classification*	*LFC*	*Std Error*	*Wilcoxon test statistic*	*Viable*	*Active*	*Adj P value*
f18c54e493d5d5ad534b9c3100216416	Proteobacteria	*Rhizobium*	1.7	0.3	-5.9	2600	2488	1.7E-06
2acee427328d2e8641b8738258b15927	Proteobacteria	*Pseudomonas*	2.3	0.3	-6.7	758	1240	1.0E-08
509fca5a90d0b1dbec313e11c762a7e1	Proteobacteria	*Rhizobium*	1.7	0.3	-5.7	384	362	8.7E-06
71582281edf705eb59a48c1a50e59a86	Proteobacteria	*Devosia*	1.6	0.3	-5.5	322	339	1.8E-05
9ca5c6b3923a29ddca908fc1a47ffca1	Proteobacteria	*Arenimonas oryziterrae*	1.4	0.2	-5.9	229	175	2.2E-06
ff8123ece2a6aff4b7f2750a928e83ec	Actinobacteriota	*Acidimicrobiales*	2.3	0.3	-7.3	210	421	1.6E-10
757d2e39206866545010c4afcc44f748	Actinobacteriota	*Micrococcaceae*	2.3	0.3	-7.7	198	362	8.4E-12
7d2d1b7d4e351f67f982eff3c2fe17cd	Verrucomicrobiota	*Chthoniobacteraceae*	2.6	0.4	-6.6	188	533	1.8E-08
1b86f9be5bce7a20bcf239e2b9d628b3	Proteobacteria	*Caulobacter*	1.6	0.3	-6.1	156	146	7.2E-07
3585021835f92062e3905fda1086a40f	Proteobacteria	*Caulobacter*	1.7	0.2	-7.1	155	179	6.2E-10
7774b77bbabdd9fdb09f14add8d48a0f	Acidobacteriota	*Vicinamibacterales*	1.4	0.2	-7.2	154	121	3.0E-10
8ad25d9768193b89b090ec9114491d2b	Acidobacteriota	*Pyrinomonadaceae*	1.3	0.3	-4.8	146	106	7.2E-04
9f9aec9e6ea1a095f866295f88499642	Acidobacteriota	*Vicinamibacterales*	1.2	0.2	-5.2	137	81	1.0E-04
56acd54962aaed6383d2cc96c94dce56	Proteobacteria	*Rhizobium*	2.5	0.3	-8.8	135	304	7.3E-16
8b10efb60e9ddd2cb7fcd09c8fa35166	Actinobacteriota	*Solirubrobacteraceae*	1.8	0.2	-10.2	122	145	1.2E-21
f6a7e105657a2c8e57f686ad1f5366f4	Acidobacteriota	*Vicinamibacterales*	1.7	0.3	-5.1	122	140	1.7E-04
6c178cce4595cdbd601c6a678885170e	Proteobacteria	*Phenylobacterium kunshanense*	1.6	0.2	-7.4	121	120	7.9E-11
c09563e31635d061641ff75dc777f7a9	Proteobacteria	*Bradyrhizobium*	1.7	0.3	-5.4	99	114	3.1E-05
05e8b422087270ed4fcfaf95cd8b94a9	Acidobacteriota	*Vicinamibacterales*	1.8	0.3	-6.8	95	106	5.2E-09
d00bdb7a37402d864b086dd2a0e7d12d	Proteobacteria	*Ramlibacter*	1.5	0.3	-5.9	69	63	2.1E-06
cf2a625547909cab32e5e208dd29cdad	Proteobacteria	*Nordella*	1.6	0.3	-5.1	68	82	1.5E-04
be42831588caa14346a2902a0790f9bb	Proteobacteria	*Sphingomonadaceae*	-2.5	0.3	9.4	64	0	3.3E-18
c9aea435821b51653cfd1fdf3162bbab	Gemmatimonadota	*Gemmatimonadaceae*	-2.4	0.2	10.0	58	0	8.1E-21
bafe930c8f8019dddab868bddc51f640	Acidobacteriota	*Thermoanaerobaculia*	1.7	0.3	-6.3	54	59	1.3E-07
721bec93178db8ac44d9855db64fd88d	Proteobacteria	*Reyranella soli*	1.5	0.2	-6.6	54	44	2.0E-08
26247a18506b8b2385fcb6460cac1500	Actinobacteriota	*Solirubrobacter soli*	2.4	0.2	-15.4	49	107	1.2E-50
7c39ad081a428ca28a5cc21dd5c874bd	Proteobacteria	*Mesorhizobium*	1.9	0.3	-5.8	45	71	5.0E-06
41b7521cb410f66d5fadd01ddfbc3d66	Cyanobacteria	*Sericytochromatia*	2.7	0.4	-6.1	45	146	7.5E-07
f13a7640696f6cda15c6e1c42b844e7e	Proteobacteria	*Sphingomicrobium*	-2.1	0.2	8.4	45	0	2.3E-14
ee49b2c458b7d8e54d794dac2230aaf1	Proteobacteria	*Mesorhizobium*	2.0	0.3	-5.9	43	64	1.7E-06
77f48eb276896ac721d9660445ccd696	Thermoproteota	*Nitrososphaeraceae*	2.4	0.4	-6.6	41	84	2.3E-08
2aaaf82eff5cd72a2d3cc69e5d4944a9	Actinobacteriota	*Ilumatobacter coccineus*	-1.9	0.2	11.5	36	0	1.1E-27
6a3b81ace6a9a68bc16486f410c773e4	Proteobacteria	*Xanthobacteraceae*	2.1	0.3	-7.9	34	56	1.3E-12
735994b04a3072f658972846a6f58909	Acidobacteriota	*Pyrinomonadaceae*	-1.6	0.2	6.8	28	0	8.5E-09
84bcc1f8b5e799a0b5fea9519520db9c	Proteobacteria	*Sphingomonas*	-1.6	0.2	6.7	27	0	1.7E-08
b2af3ab5d31f6e0388d7e26fcb0b54d0	Actinobacteriota	*Acidimicrobiales*	-1.5	0.3	5.0	27	0	2.7E-04

daac2813ec4defb7cdc75147d26333bc	Verrucomicrobiota	*Verrucomicrobiaceae*	3.1	0.6	-4.8	19	50	9.7E-04
4d2718a6cb2774d782d35b0fb3f43e58	Cyanobacteria	*Sericytochromatia*	4.5	0.8	-5.3	16	131	6.7E-05
0f995b24faa7fd071914de1aefaf880a	Firmicutes_D	*Planococcaceae*	3.9	0.7	-5.3	14	63	8.6E-05
0669f23d503b4ff7ff013e65bc4ecb67	Proteobacteria	*Micavibrio*	3.4	0.7	-5.1	6	25	1.8E-04
a6facc90b4326eef8109db25acb11d38	Proteobacteria	*Xanthobacteraceae*	3.8	0.8	-4.8	0	17	8.1E-04
69f0863c21ffb4985a0586fdbfbd564c	Proteobacteria	*Pseudolabrys*	3.7	0.6	-6.0	0	13	1.0E-06
4e893d68fbf03b0af9022a974dcb9265	Planctomycetota	*Tepidisphaeraceae*	4.2	0.9	-4.8	0	26	8.9E-04
6870e333679b726bbb8993ea7b1c832f	Acidobacteriota	*Vicinamibacteria*	4.3	0.8	-5.2	0	25	1.4E-04
2a13b4250e06e6fc3980788942bf86dc	Proteobacteria	*Dongiaceae*	3.9	0.7	-5.2	0	16	1.3E-04
61f1dfd764ba55f5a494b3f5337141a8	Proteobacteria	*Burkholderiaceae*	3.7	0.8	-4.9	0	13	5.8E-04
bb535db5a56df06eb50a972e6824b907	Proteobacteria	*Usitatibacteraceae*	4.0	0.7	-6.1	0	18	5.8E-07

Supplemental Table 9: Results of binomial generalized linear models of successful plant colonization. Results from 1) null model 2) model using ASV abundance in the Viable fraction as a predictor 3) model using ASV abundance in the Active fraction as a predictor. Estimates and Std. Error are reported as untransformed log-odds.

Null Model: presence in plant(1,0) ~ replicate plant
AIC: 600.32
Null deviance: 593.82 on 2467 degrees of freedom

Predictor	*Estimate*	*Std. Error*	*Z value*	*P value*
Intercept	*-3.56E+00*	*2.46E-01*	*-14.49*	*<2e-16*
Rep2	*5.88E-02*	*3.43E-01*	*0.171*	*0.864*
Rep3	*1.17E-13*	*3.48E-01*	*0*	*1*
Rep4	*-3.57E-01*	*3.81E-01*	*-0.935*	*0.35*

Model 2: presence in plant(1,0) ~ abundance in rhizosphere viable +replicate plant
AIC: 572.01
Null deviance: 593.82 on 2467 degrees of freedom

Predictor	*Estimate*	*Std. Error*	*Z value*	*P value*
Intercept	*-3.68E+00*	*0.2482*	*-14.85*	*2.00E-16*
Abundance Viable	*1.93E-03*	*0.0004*	*5.18*	*2.27E-07*
Rep2	*-1.05E-02*	*0.3512*	*-0.03*	*0.976*
Rep3	*-7.20E-02*	*0.3545*	*-0.20*	*0.839*
Rep4	*-4.30E-01*	*0.3900*	*-1.10*	*0.27*

Model 3: presence in plant(1,0) ~ abundance in rhizosphere active
AIC: 555.46
Null deviance: 593.82 on 2467 degrees of freedom

Predictor	*Estimate*	*Std. Error*	*Z value*	*P value*
Intercept	*-3.85E+00*	*0.2672*	*-14.42*	*2.00E-16*
Abundance Active	*3.53E-03*	*0.0007*	*4.98*	*6.43E-07*
Rep2	*1.06E-01*	*0.3598*	*0.30*	*0.767*
Rep3	*3.49E-02*	*0.3650*	*0.10*	*0.924*
Rep4	*-3.59E-01*	*0.4013*	*-0.89*	*0.371*

Supplementary Table 10: Ten most abundant successful plant colonizers in the rhizosphere active fraction.

ASVs	***Phyla***	***Lowest taxonomic classification***	***Mean active***	***SD active***	***Mean viable***	***SD viable***
f18c54e493d5d5ad534b9c3100216416	*Proteobacteria*	*Rhizobium*	2333.8	131.7	2451.5	1053.3
2acee427328d2e8641b8738258b15927	*Proteobacteria*	*Pseudomonas*	1022.3	516.7	1084.8	582.3
71582281edf705eb59a48c1a50e59a86	*Proteobacteria*	*Devosia*	364.5	10.6	326.5	43.1
509fca5a90d0b1dbec313e11c762a7e1	*Proteobacteria*	*Rhizobium*	347.0	43.7	347.3	149.3
e2b0e8123407c0522e65cc247a76628f	*Proteobacteria*	*Rhizobiaceae*	287.0	NA	56.0	NA
a799adf1c40ad36daa84625bd855e8ee	*Proteobacteria*	*Variovorax paradoxus*	249.0	44.7	188.0	31.4
3585021835f92062e3905fda1086a40f	*Proteobacteria*	*Caulobacter*	216.7	46.3	162.3	13.7
3e37cbf592eed8db558988be92d6cc49	*Firmicutes_D*	*Neobacillus*	189.5	181.7	70.5	40.3
1b86f9be5bce7a20bcf239e2b9d628b3	*Proteobacteria*	*Caulobacter*	173.0	NA	172.0	NA
d44ee1c36fcc9b098d56b04791f30386	*Proteobacteria*	*Pseudomonas*	156.5	89.7	165.3	83.0
